# Road Performance of Hot Central Plant Versus Hot In-Place Recycling Asphalt Mixtures: A Quantitative Comparison and Adaptability Analysis

**DOI:** 10.3390/ma18225149

**Published:** 2025-11-12

**Authors:** Qinyu Shi, Lixin Zhou, Bo Li

**Affiliations:** 1 School of Digital Construction, Shanghai Urban Construction Vocational College, Shanghai 200438, China; 2 College of Civil Engineering and Transportation, Yangzhou University, Yangzhou 225127, China

**Keywords:** asphalt mixture, hot central plant recycling, hot in-place recycling, basalt fibers, road performance

## Abstract

Despite being crucial for sustainable pavement construction, the widespread application of hot recycled asphalt mixtures in high-grade surface courses is hindered by concerns over their long-term performance, particularly regarding cracking resistance and moisture stability. This study systematically evaluates the road performance of hot central plant recycling (HCPR with 30% RAP) and hot in-place recycling (HIPR with 80% RAP) mixtures, benchmarked against virgin hot mix asphalt (SMA-13), through comprehensive laboratory simulations. The enhancing effect of basalt fibers (BFs) was thoroughly investigated. Results revealed a significant performance trade-off; while the recycled mixtures exhibited superior high-temperature stability (e.g., an 80.7% increase in dynamic stability for HIPR), their cracking resistance substantially decreased with higher RAP content (e.g., reductions of 60.8% in low-temperature flexural strain and 22.1% in intermediate-temperature fracture energy for HIPR). Both recycled mixtures also showed susceptibility to moisture damage, evidenced by stripping in Hamburg wheel-tracking tests. The incorporation of BFs effectively mitigated these deficiencies. It comprehensively improved the performance, enabling the HCPR mixture to meet specifications for severely cold regions and elevating the HIPR mixture to compliance level for cold regions. Furthermore, BF significantly enhanced rutting resistance under coupled hydrothermal conditions. These findings demonstrate that basalt fiber reinforcement can bridge the performance gap of recycled mixtures, thereby expanding their application scope and providing a robust technical basis for selecting and optimizing recycling strategies in high-grade pavement engineering.

## 1. Introduction

According to statistics, approximately 12% to 15% of high-grade asphalt pavements require annual repair and maintenance. Consequently, the annual output of RAP from milling and overlay works in China reaches no less than 200 million tons [1]. Discarding these aged asphalt mixtures not only causes environmental pollution but also represents a significant waste of resources. Against the backdrop of the “Dual Carbon” goals, the rational utilization of RAP has become a crucial pathway for green development in road engineering.

Hot central plant and hot in-place recycling are two widely used hot recycling technologies for RAP in current engineering practice. The process of hot central plant recycling asphalt mixture involves milling the existing asphalt pavement, conveying the milled material to a mixing plant, and mixing it with new materials following a designed gradation to manufacture a recycled mixture for pavement construction [2,3]. This technology is characterized by its controllable process and applicability to various pavement structural layers, leading to its extensive application [4]. Hot in-place recycling utilizes specialized equipment to heat and scarify the in-service pavement in situ [5]. Based on laboratory tests, the required proportions of rejuvenator and new asphalt mix are determined: these components are then uniformly mixed in a remixer truck before final compaction [6]. This method offers advantages such as potentially 100% RAP utilization, rapid construction speed, and minimal traffic disruption, contributing to its growing adoption. Compared to traditional hot mix asphalt, hot recycling technologies demonstrate significant advantages in terms of energy consumption, material usage, and cost-effectiveness [7]. Research shows that when the RAP content in hot central plant recycling asphalt mixture is between 20% and 30%, the CO_2_ emissions during its production are merely 80% of those of new hot mix asphalt, and the construction cost can be reduced by about 30% [8]. For hot in-place recycling asphalt mixture, which utilizes 100% RAP with only 10–20% new mixture for gradation adjustment, the carbon emissions are reported to be 49% of those from new hot mix asphalt, and project costs can be reduced by about 30% to 50% [9,10].

Despite the evident economic and environmental benefits of hot recycling technologies, management authorities remain cautious about their large-scale application in surface layers of high-grade pavements. This caution stems primarily from concerns regarding the performance deficiencies of recycled pavements. Research shows that while the road performance of hot central plant recycling mixture generally meets specifications or design requirements, its crack resistance still lags significantly behind that of hot mix asphalt [11,12], rendering them more susceptible to cracking, raveling, and spalling [13]. Owing to the greater proportion of RAP, the crack resistance and other properties of hot in-place recycling mixtures degrade further, performing worse than hot central plant recycling mixtures [14,15]. These conclusions are drawn from studies conducted by different researchers across various regions, utilizing RAP from diverse sources. However, for specific road sections, a systematic and quantitative comparative study on the performance differences between mixtures corresponding to different maintenance strategies—namely, milling and repaving with new material, hot central plant recycling mixture, or hot in-place recycling mixtures—remains lacking.

Furthermore, previous studies have indicated that incorporating fibers into hot recycled asphalt mixtures can effectively enhance their overall road performance [16]. Specifically, the addition of basalt fibers has been shown to significantly improve the resistance to low-temperature brittleness, fatigue resistance, and freeze–thaw durability of recycled mixtures. Notably, under heavy traffic conditions, basalt fibers effectively inhibit the initiation and propagation of cracks [17,18].

However, most existing studies focus on a single recycling technology. A systematic and quantitative comparison of the performance differences between HMA, HCPR, and HIR mixtures, especially under identical laboratory conditions and with the same RAP source, is still insufficient. Moreover, the potential of basalt fibers to mitigate the performance deficiencies of both recycling methods has not been fully explored and quantified. This study aims to quantitatively evaluate and compare the pavement performance of HMA, HCPR (with 30% RAP), and HIPR (with 80% RAP) mixtures through a series of laboratory tests. The specific objectives include: (1) to assess the resistance to permanent deformation (high-temperature performance), resistance to thermal cracking (low-temperature performance), resistance to intermediate-temperature cracking, and water susceptibility; (2) to investigate the enhancing effects of basalt fibers on these asphalt mixtures; (3) to conduct an adaptability analysis of these recycling technologies for highway applications. The findings of this research aim to provide a performance-based rationale for the application of hot recycling technologies in asphalt pavements, thereby promoting the efficient utilization of resources and facilitating the green transformation of road engineering.

## 2. Materials and Mixture Preparation

### 2.1. Aggregates and Asphalt

Basalt was selected as the coarse aggregate in this research, and limestone was adopted for both fine aggregates and mineral fillers. The asphalt binder utilized was PG 76-22 SBS-modified asphalt. All constituent materials satisfied all specified requirements in [19].

### 2.2. Reclaimed Asphalt Pavement (RAP)

The Reclaimed Asphalt Pavement (RAP) material used in this laboratory study was sourced exclusively from a single rehabilitation project of the Shanghai–Nanjing Expressway and was homogenized through the quartering method to ensure consistency. Experimental tests were conducted to measure the performance parameters of the extracted aged asphalt binder [18], with the results compiled in Table 1. The homogeneity of the RAP was confirmed by the low variability in test results, as reflected by error bars within approximately 10% in relevant data. As shown, the penetration and ductility of the aged binder are lower than the requirements for virgin SBS-modified asphalt specified in [19], which is indicative of aging that has caused the binder to become harder and more brittle. Furthermore, according to the aging classification method in reference [20], the degree of aging of this reclaimed binder can be classified as Level II aging. The sieving results of RAP aggregates are shown in Table 2, the gradation of the aggregates in the RAP still fell within the specified limits for SMA-13 mixtures.

### 2.3. Rejuvenator

The RA-102 rejuvenator was employed in this study. Its technical properties were evaluated following the Chinese specification JTG/T 5521-2019 [21] (Technical Specification for Highway Asphalt Pavement Recycling). All tested indicators satisfied the requirements. Key properties included a viscosity of 4000 cP at 90 °C, with saturates and aromatics contents of 25.6% and 53%, respectively.

### 2.4. Fibers

The properties of the chopped basalt fibers and lignin fibers used in this investigation are summarized in Table 3 and Table 4, respectively. All tested properties conformed to the requirements of the relevant specifications.

### 2.5. Mix Design

Since the RAP was still of SMA-13 gradation, this study maintained this mix design for preparing the recycled mixtures. Drawing on prior research findings, the RAP (reclaimed asphalt pavement) content was specified as 30% for the hot central plant recycling (HCPR) mixture and 80% for the hot in-place recycling (HIR) mixture. Conventional hot mix asphalt served as the control group. The optimum asphalt contents for the different mixtures were determined using the Marshall mix design method. The criteria for determining the optimum content were based on a comprehensive consideration of Marshall stability, flow value, and volumetric parameters (air voids, VMA, and VFA) as per the Chinese specification JTG F40-2004 [19]. The final mix proportions used in the testing program are presented in Table 5.

### 2.6. Preparation of Hot Recycled Asphalt Mixtures

(1) Preparation of Hot Central Plant Recycling Asphalt Mixture

**Figure 1:**
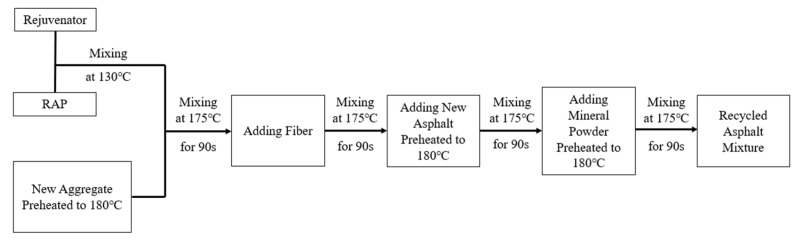
Laboratory simulation of Hot Central Plant Recycling process.

Following the construction process of hot central plant recycling, the laboratory simulation of the preparation procedure is illustrated in Figure 1. During this process, the RAP was preheated to 130 °C, consistent with the heating temperature in the plant recycling drum.

(2) Preparation of Hot In-Place Recycling Asphalt Mixture

**Figure 2:**
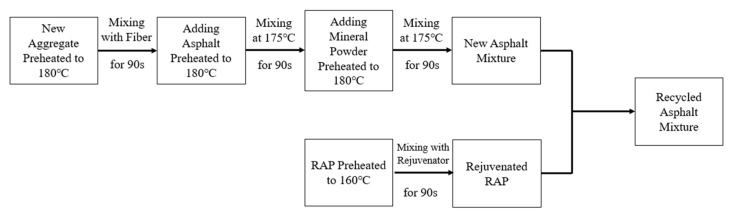
Laboratory simulation of Hot In-Place Recycling process.

The laboratory fabrication procedure, simulating the field construction procedure for hot in-place recycling, is shown in Figure 2. The RAP material was preheated and maintained at 160 °C, corresponding to the typical heating temperature used in field operations.

(3) Determination of Rejuvenator Content

A performance-oriented design method was used to confirm the optimum dosage of rejuvenator. The initial dosages were set as 4%, 6%, 8%, and 10% based on the mass of the aged asphalt binder in RAP. Subsequently, the performance of the recycled asphalt was tested after incorporating these different dosage levels, with the results presented in Table 6. As presented in Table 6, when the rejuvenator content reached 6%, both of the penetration value and softening point value of the recycled asphalt had been substantially restored to levels comparable to those of the virgin SBS-modified asphalt binder. Consequently, the optimal rejuvenator content was established at 6% by mass of the aged asphalt in the RAP.

## 3. Test Methods

### 3.1. Wheel Tracking Test

The wheel tracking test was performed in line with the T0719 standard specified in China’s JTG E20 [23]. During the test, the temperature was controlled at 60 °C and the applied load was 0.7 MPa. Permanent deformation of high-temperature was evaluated using the dynamic stability (DS) index, where a higher DS value indicates superior resistance to permanent deformation.

### 3.2. Dynamic Creep Test

The dynamic creep test was performed following the NCHRP 9-29 protocol using a UTM-25 servohydraulic testing system (IPC Global Co., Melbourne, Australia). Testing was carried out at 60 °C with an applied stress of 0.7 MPa. The loading pattern consisted of a 0.1 s haversine load pulse followed by a 0.9 s rest period. The test was terminated either upon reaching 100,000 microstrains or after 10,000 load cycles. High-temperature performance was assessed using the flow number (FN), with higher values denoting better resistance to rutting.

### 3.3. Low-Temperature Bending Beam Test

This test was conducted following the standardized procedure T0715 in JTG E20, and the test was conducted at −10 °C. The failure strain was used to characterize the resistance to low-temperature cracking performance, and larger strain values mean better resistance to thermal cracking.

### 3.4. Semi-Circular Bend (SCB) Test

The SCB test was performed following AASHTO TP 105-13 [24] at a temperature of 25 °C. Fracture energy (Gf) and Flexibility Index (FI) were used to evaluate intermediate-temperature cracking resistance [25], with higher values of both parameters representing improved crack resistance.

### 3.5. Freeze–Thaw Splitting Test

The freeze–thaw splitting test was executed according to T0729 of JTG E20. Moisture susceptibility was quantified by the tensile strength ratio (TSR), where a higher TSR value corresponds to enhanced resistance to moisture-induced damage.

### 3.6. Hamburg Wheel-Tracking Test

**Figure 3:**
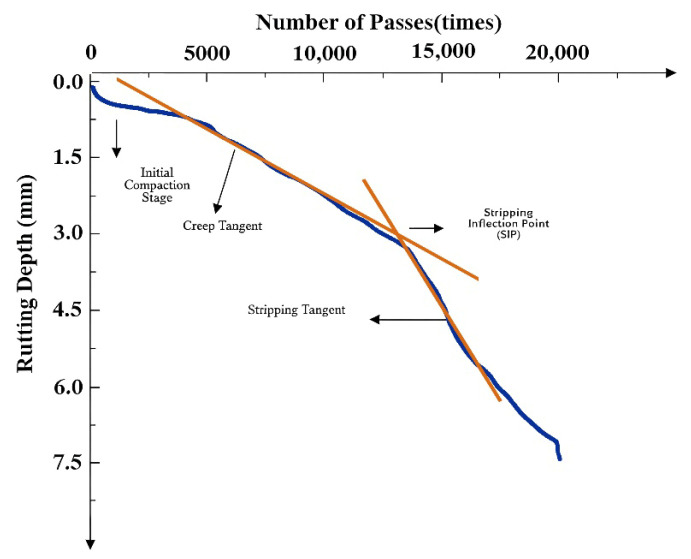
Typical Hamburg wheel-tracking test curve.

The Hamburg wheel-tracking test was conducted following AASHTO T324 specifications [26]. The test was conducted in a 50 °C water bath to simulate combined moisture and temperature conditions, evaluating both high-temperature performance and moisture damage resistance. A typical test result is shown in Figure 3, where the curve can be divided into three stages: initial compaction, creep, and stripping. A decrease in the creep slope indicates a reduced deformation rate during the creep phase, reflecting improved high-temperature stability. The intersection point of the fitted creep and stripping stage curves is identified as the stripping inflection point. A higher position of this point, indicating a greater number of load passes reached before stripping, reflects enhanced resistance to moisture damage and better overall moisture stability. Furthermore, a reduced slope in the stripping stage also signifies improved resistance to moisture-induced stripping. The test was terminated after 20,000 loading cycles.

All asphalt mixtures were prepared in accordance with the Chinese specification JTG 3410-2025 [27]. For each mixture type and testing condition, three replicate specimens were fabricated and tested. The reported results represent the average value of these three replicates.

## 4. Results and Analysis

### 4.1. High-Temperature Stability Performance

(1) Wheel Tracking Test Results

**Figure 4:**
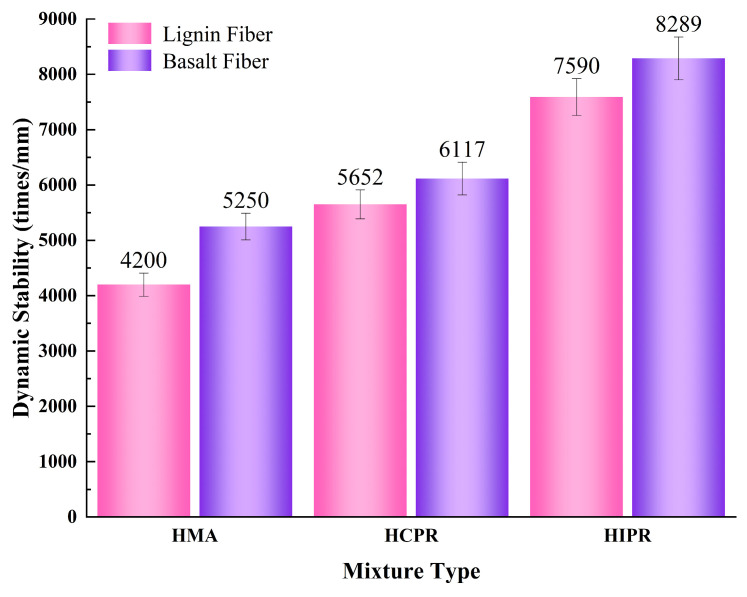
Results of Wheel Tracking Test.

The results of the wheel tracking test for different asphalt mixtures are shown in Figure 4. Figure 4 shows that the high-temperature stability of hot recycled asphalt mixtures was significantly better than that of virgin hot mix asphalt. For the mixtures with lignin fiber, the dynamic stability of the hot central plant recycling (HCPR) mixtures increased by 34.6%. In contrast, the hot in-place recycling asphalt mixture achieved a more significant improvement of 80.7% when compared to the virgin mixtures. The greater enhancement in dynamic stability for the in-place recycled mixture is primarily attributed to its higher RAP content. The increased proportion of aged asphalt in the mixture results in greater overall hardness and enhanced resistance to high-temperature shear deformation [28].

Furthermore, the incorporation of basalt fibers positively influenced the high-temperature stability of the recycled asphalt mixtures. Specifically, for the virgin, hot central Plant Recycling Mixtures, and Hot In-Place Recycling Mixtures, the addition of basalt fibers increased the dynamic stability by 25%, 8.2%, and 9.2%, respectively, compared to their counterparts using lignin fibers. It is noteworthy that the improvement in high-temperature performance due to basalt fibers was less pronounced in the recycled mixtures than in the virgin mixture. This can be attributed to the fiber-introduced interfaces, which absorb free asphalt, thereby increasing the mixture’s viscosity [29]. However, in hot recycled mixtures, the asphalt has already undergone an aging process, resulting in higher viscosity and a lower content of free asphalt, which thereby limits the viscosity-enhancing effect of the basalt fibers.

(2) Dynamic Creep Test Results

**Figure 5:**
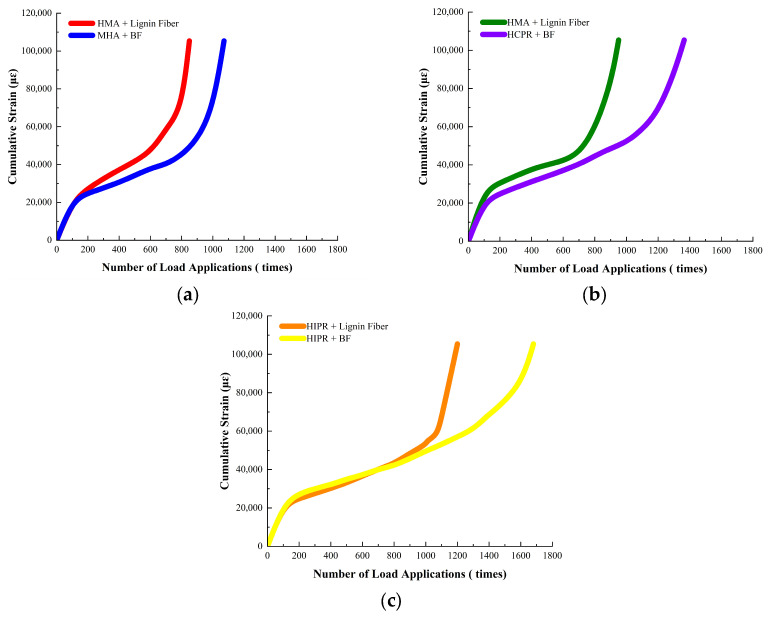
Cumulative strain curves from Dynamic Creep Test. (**a**) HMA (**b**) HCPR (**c**) HIPR.

The cumulative strain curves of different asphalt mixtures under dynamic creep loading are presented in Figure 5, while key parameters, including the creep rate and flow number (Fn), are summarized in Table 7.

It can be observed from Figure 5 and Table 7 that hot recycled asphalt mixtures had notably stronger resistance to high-temperature creep than virgin asphalt mixtures. For example, in the mixtures modified with lignin fibers, the creep rates of hot central plant recycled mixtures and hot in-place recycled mixtures dropped by 39.1% and 52.2%, respectively, while their flow numbers increased by 41.7% and 77.5% compared to the virgin mixtures. These findings are consistent with the dynamic stability results.

Furthermore, basalt fiber incorporation demonstrated beneficial effects in improving the high-temperature creep resistance across all mixture types. Compared to mixtures containing lignin fiber, the use of basalt fibers increased the flow number by 23.1% to 35.5% across the evaluated mixture types.

### 4.2. Low-Temperature Crack Resistance

**Figure 6:**
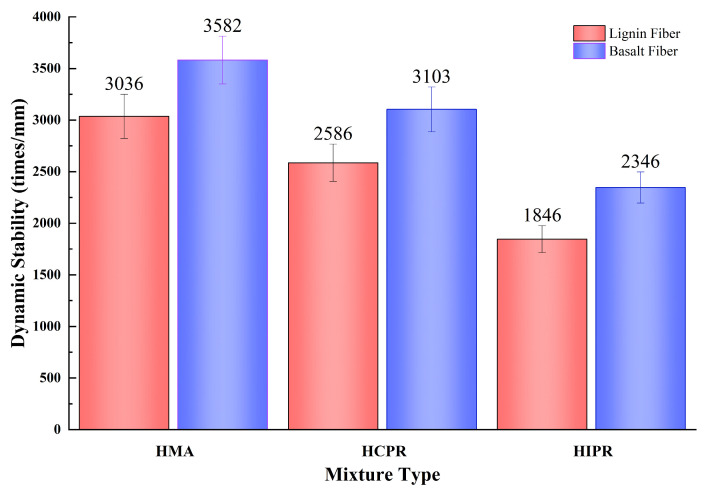
Results of low-temperature bending beam test.

The results of the low-temperature bending beam tests for the different asphalt mixtures are presented in Figure 6. As shown, the resistance to low-temperature cracking of the hot recycled mixtures was markedly inferior to that of the virgin mixture. Taking the lignin fiber-modified mixtures as an example, compared to the virgin mixture, the maximum flexural tensile strains of the hot central plant recycled and hot in-place recycled mixtures decreased from 3036 µε to 2586 µε and 1846 µε, representing reductions of 14.8% and 60.8%, respectively. According to the Chinese specification JTG F40 [19], the virgin mixture qualifies for use in extremely cold regions (requirement: ≥2600 µε), the plant-mixed recycled mixture is suitable for cold regions (requirement: ≥2300 µε), whereas the in-place recycled mixture fails to meet the requirement for both cold and temperate winter regions (≥2000 µε). This performance degradation is attributed to the reduction in aromatics and saturates within the aged asphalt binder, resulting in embrittlement and a consequent loss of mixture toughness. Although the rejuvenator partially restored the asphalt properties, its effectiveness in improving low-temperature performance was limited.

In contrast, the incorporation of basalt fibers significantly enhanced the maximum flexural tensile strain of all asphalt mixtures. Compared to their lignin fiber-modified counterparts, the virgin, hot central plant recycled, and hot in-place recycled mixtures containing basalt fibers exhibited increases in maximum flexural tensile strain of 18.0%, 20.0%, and 27.1%, respectively. Notably, the hot central plant recycling mixture with basalt fibers met the requirement for extremely cold regions (≥2600 µε), an upgrade from initially only satisfying the cold region requirement. Furthermore, the hot in-place recycling mixture with basalt fibers achieved compliance with the cold region specification. This improvement is ascribed to the basalt fibers, which disperse within the asphalt mixture to form a robust network structure that effectively redistributes stress and provides reinforcement, thereby enhancing crack resistance [30].

### 4.3. Intermediate-Temperature Crack Resistance

**Figure 7:**
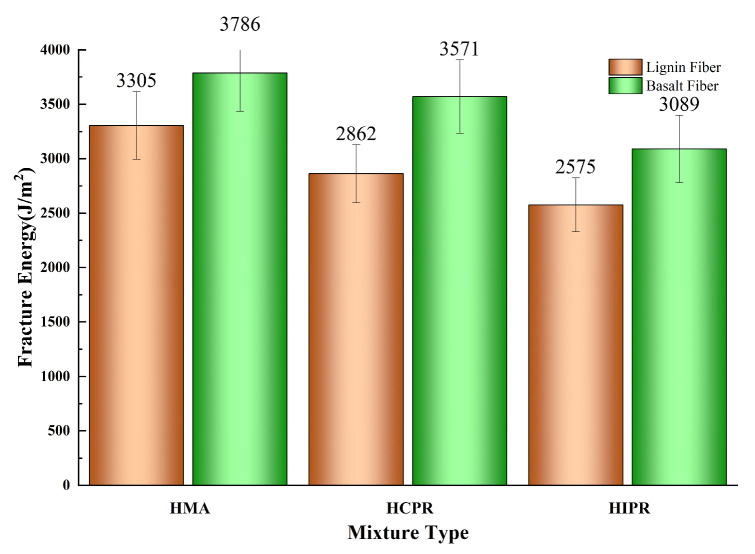
Results of fracture energy (Gf).

**Figure 8:**
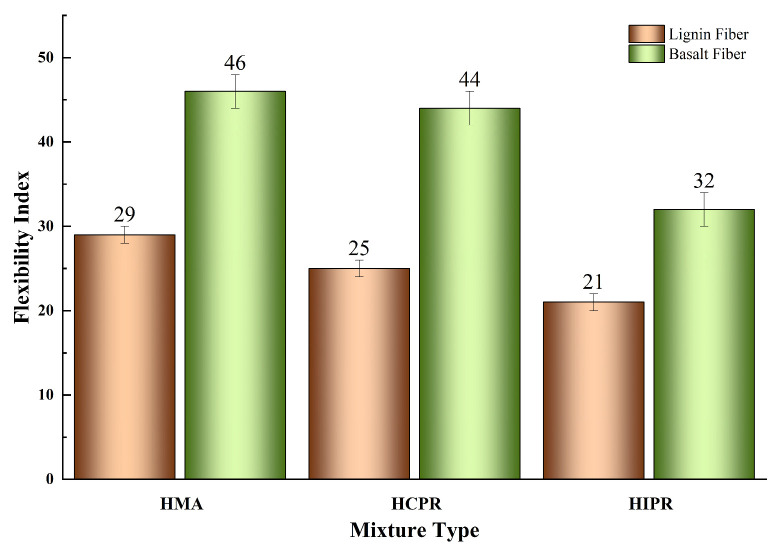
Results of Flexibility Index (FI).

The semi-circular bending (SCB) test results for various asphalt mixtures are shown in Figure 7 and Figure 8. The data reveal that the resistance to intermediate-temperature cracking of the hot recycled mixtures is inferior to that of the virgin mixture. For instance, in the lignin fiber-modified mixtures, the fracture energy (Gf) of the hot central plant recycling and hot in-place recycling mixtures decreased from 3305 J/m^2^ in the virgin mixtures to 2861 J/m^2^ and 2575 J/m^2^, representing reductions of 13.4% and 22.1%, respectively. Correspondingly, the Flexibility Index (FI) values decreased by 13.8% and 27.6%. These results demonstrate that higher RAP content leads to more significant adverse effects on the crack resistance of recycled mixtures. This phenomenon is attributed to the aged, brittle, and hardened characteristics of the RAP material in the recycled mixtures, which impair the overall relaxation capacity and ductility of the mixture. Consequently, particular attention should be given to enhancing the crack resistance of mixtures produced using the hot in-place recycling method. This finding was consistent with previous research documented in the literature [31].

Furthermore, the incorporation of basalt fibers substantially enhanced the intermediate-temperature cracking resistance across all mixture types. Compared with lignin fiber-modified mixtures, the addition of basalt fibers increased the fracture energy (Gf) of virgin, hot central plant recycling, and hot in-place recycling mixtures by 14.5%, 24.7%, and 20.0%, respectively. This improvement indicates that basalt fibers enhance the energy absorption capacity of mixtures during cracking, thereby improving crack resistance. Correspondingly, the Flexibility Index (FI) values increased by 58.6%, 76.0%, and 52.4%, demonstrating that basalt fibers effectively retard crack propagation rates. This enhancement is attributed to the ability of basalt fibers to disperse and transfer loads within the mixture, resulting in more uniform stress distribution, reduced stress concentration, and consequently, improved resistance to crack propagation [32]. Notably, with basalt fiber addition, both Gf and FI of the hot central plant recycling mixture exceeded those of the virgin asphalt mixture. Meanwhile, the Gf of the hot in-place recycling mixture approached that of the virgin mixture, with its FI also showing a significant improvement. These findings confirm that basalt fiber incorporation can significantly improve the cracking resistance of hot recycled asphalt mixtures, thereby expanding their potential application scope.

### 4.4. Moisture Susceptibility

**Figure 9:**
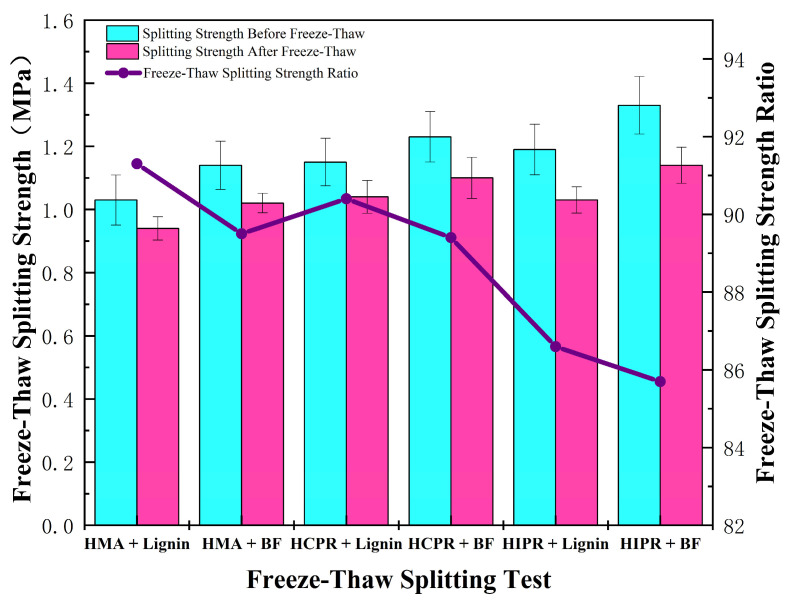
Results of freeze–thaw splitting test.

Figure 9 shows the results of the freeze–thaw splitting test for different asphalt mixtures. It can be seen that the moisture resistance of hot recycled mixtures was somewhat lower than that of virgin mixtures. For the lignin fiber-modified mixtures, the hot central plant recycling mixture showed a modest reduction in Tensile Strength Ratio (TSR), while the hot in-place recycling mixture demonstrated a more pronounced decrease of 4.7 percentage points relative to the virgin mixture. This indicates that higher RAP content adversely affects the moisture stability of recycled mixtures, although all measured TSR values still satisfied the specification requirement of ≥80%.

Compared to mixtures containing lignin fibers, those incorporating basalt fibers exhibited comparable TSR values. However, the absolute splitting strengths, both before and after freeze–thaw conditioning, increased by 7.0% to 14.0% across the mixture types. This enhancement is primarily attributed to the dispersion of basalt fibers within the mixture, where they acted similarly to reinforcement, promoting stress distribution and shared load-bearing within the material [33].

### 4.5. Performance Under Simulated Moisture–Thermal Conditions

**Figure 10:**
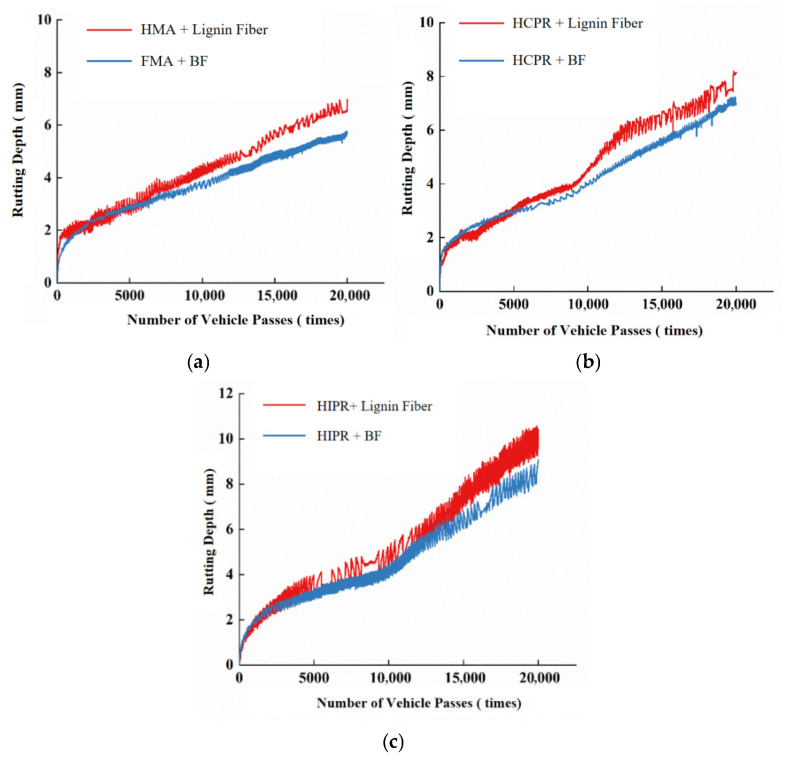
Hamburg wheel-tracking test curves. (**a**) HMA. (**b**) HCPR. (**c**) HIPR.

The Hamburg wheel-tracking test results for the different asphalt mixtures are presented in Figure 10. The corresponding performance indicators calculated from these tests are summarized in Table 8.

Analysis of Figure 10 and Table 8 reveals that the rutting depth curve of the virgin asphalt mixture remained within the creep stage throughout the test, without entering the stripping stage. In contrast, the curves for both the HCPR and HIPR mixtures progressed into the stripping stage. These results demonstrate that the resistance to high-temperature deformation and moisture-induced stripping was compromised for hot recycled mixtures in immersed conditions. Taking lignin fiber-modified mixtures as an example, the maximum rutting depths of the hot central plant recycling and hot in-place recycling mixtures increased from 6.91 mm to 8.15 mm and 10.03 mm, respectively. The creep rates increased by 38.5% for the HCPR mixture and 100% for the HIPR mixture, while the stripping inflection points occurred at 12,483 and 8637 load cycles, respectively, compared to the virgin mixture. These findings indicate that both types of recycled mixtures experienced reduced performance against combined thermal and moisture damage under simulated rain-thermal conditions, with the hot in-place recycling mixture showing more pronounced deterioration.

Furthermore, the incorporation of basalt fibers substantially enhanced the resistance to high-temperature deformation and moisture-induced stripping across all mixture types. Compared to lignin fiber-modified mixtures, the addition of basalt fibers reduced creep rates by 16.7% and 15.4% in hot central plant recycling and hot in-place recycling mixtures, respectively, while increasing the stripping inflection points by 25.2% and 22.0%. This improvement mechanism can be attributed to the network of dispersed, high-strength, high-modulus basalt fibers throughout the mixture, forming a reinforcing network that effectively distributes stress and stabilizes deformation under repeated loading [34].

### 4.6. Discussion

Compared with virgin asphalt mixture, the two types of hot recycled mixtures exhibit distinct performance characteristics. As for high-temperature stability, the recycled mixtures demonstrate significant advantages, with the hot in-place recycling mixture showing the most pronounced improvement in dynamic stability (80.7% increase). This enhancement is primarily attributed to its high RAP content (80%), which introduces more aged asphalt, thereby increasing the mixture’s stiffness and shear resistance. This is consistent with the research findings in the reference [19]. However, the resistances to intermediate- and low-temperature cracking of the recycled mixtures are notably inferior. The low-temperature flexural tensile strain of the hot central plant recycling (RAP 30%) and hot in-place recycling (RAP 80%) mixtures decreased by 14.8% and 60.8%, respectively, while their intermediate-temperature fracture energy reduced by 13.4% and 22.1%. This indicates that as RAP proportion increases, the embrittlement of aged asphalt compromises its inherent toughness and presents progressively worse cracking resistance. This is consistent with the research conclusion in the reference [29]. Regarding moisture susceptibility, although all recycled mixtures meet specification requirements, both exhibited stripping in the Hamburg wheel-tracking test that simulates actual moisture–thermal conditions, indicating their moisture damage resistance remains inferior to that of virgin mixture in realistic hydrothermal environments. The study in reference [11] also made the same finding.

Based on these performance differences, the hot central plant recycling mixture (RAP 30%) exhibits more balanced overall performance. While maintaining good high-temperature stability, its low- and intermediate-temperature cracking resistance, restored by the rejuvenator, can meet the requirements for intermediate and base courses of high-grade pavements in most regions of China (e.g., cold regions). The hot in-place recycling mixture (RAP 80%), with its excellent high-temperature rutting resistance, is more suitable for areas with heavy traffic loads and prolonged high summer temperatures. However, due to its relatively poor cracking resistance, its application is recommended primarily in regions with less stringent low-temperature cracking requirements.

Furthermore, compared to conventional lignin fiber reinforcement, BF modification achieves superior performance enhancement: increasing dynamic stability by 28.9–41.7%, improving flexural tensile strain by 35.8–52.1%, and elevating the freeze–thaw tensile strength ratio by 8.3–13.8%. These quantitative improvements highlight BF’s exceptional capability in enhancing both high-temperature stability and low-temperature crack resistance simultaneously. It indicates that incorporating basalt fibers comprehensively addresses the performance limitations of various asphalt mixtures, effectively expanding the application scope of both recycling technologies. For the hot central plant recycling mixture, the addition of BF enables its low-temperature performance to meet technical requirements for extremely cold regions (specification requirement: ≥2800 με), while its intermediate-temperature cracking resistance (with a fracture energy of 8.9 kJ/m^3^) even surpasses that of conventional virgin mixture. This enhancement makes the modified hot central plant recycling mixture potentially suitable for pavement structures in severe cold regions. For the hot in-place recycling mixture, fiber incorporation elevates its low-temperature performance from “non-compliant” to meeting cold region requirements, while further consolidating its high-temperature stability (dynamic stability increased from 6525 to 12,500 cycles/mm)and hydrothermal stability (freeze–thaw tensile strength ratio improved from 78.2% to 89.0%). This significantly enhances the reliability of hot in-place recycling technology with high RAP content, establishing it as a competitive rapid maintenance solution applicable under broader geographical and more demanding traffic conditions.

From a material cost perspective, using basalt fibers in SMA-13 mixtures increases the cost by approximately 48.6 CNY per ton, along with a one-time equipment investment of 80,000 CNY. This cost increment primarily results from the price difference between basalt fibers (22,000 CNY/ton) and lignin fibers (4200 CNY/ton). Despite the higher initial cost, considering long-term performance, this study confirms that basalt fibers provide 30–50% greater improvement in rutting resistance, crack resistance, and durability compared to traditional lignin fibers. This performance advantage will translate into extended pavement service life and reduced long-term maintenance requirements. Therefore, although basalt fibers increase initial costs, their long-term performance benefits effectively balance the initial investment, demonstrating better overall economy throughout the entire life cycle.

## 5. Conclusions

(1) Hot recycled asphalt mixtures demonstrate a distinct “high-strength, low-toughness” characteristic compared to virgin HMA. Quantitatively, the high-temperature stability, as measured by dynamic stability, significantly increases, with the HIPR mixture (80% RAP) showing the most pronounced improvement of 80.7% over the virgin mix. Conversely, cracking resistance deteriorates markedly with higher RAP content. The HIPR mixture exhibits a 60.8% reduction in low-temperature flexural tensile strain and a 22.1% decrease in intermediate-temperature fracture energy compared to the virgin mix, representing substantially server deterioration than the HCPR mixture (30% RAP).
(2) While the freeze–thaw splitting strength ratio of all recycled mixtures met the specification requirement (≥80%), their performance in the Hamburg wheel-tracking test revealed limitations. Both HCPR and HIPR mixtures exhibited clear stripping behavior under simulated hydrothermal conditions, with stripping inflection points recorded at 12,483 and 8637 load cycles, respectively. This indicates a potential vulnerability to moisture damage in real-world, rainy and hot environments that is not captured by conventional tests.
(3) The incorporation of basalt fibers (BFs) effectively mitigates the performance deficiencies of recycled mixtures. The three-dimensional network formed by the fibers delivers comprehensive enhancements: it increases dynamic stability by 8.2–9.2%, boosts low-temperature flexural tensile strain by 20.0–27.1%, and raises the fracture energy by 20.0–24.7% compared to lignin fiber-reinforced counterparts. This synergistic improvement makes BF a superior modifier for tackling the performance trade-offs in recycling.
(4) Based on performance balance and engineering applicability, hot central plant recycling mixture (RAP 30%) is recommended for surface courses of high-grade pavements in most regions of China. Meanwhile, hot in-place recycling mixture (RAP 80%), with its exceptional high-temperature rutting resistance, is recommended for applications in regions less sensitive to low-temperature cracking.
(5) As an efficient reinforcing material, basalt fibers effectively compensate for the performance deficiencies of hot recycled mixtures. Their incorporation enables the application of hot central plant recycling mixtures to be extended to severely cold regions. The reliability of hot in-place recycling mixtures is also significantly enhanced, establishing them as viable rapid maintenance solutions under broader climatic and traffic conditions. This advancement strongly promotes the high-quality and sustainable development of asphalt pavement recycling technology.

### Limitations and Future Research

This study was conducted under controlled laboratory conditions using RAP from a single source. Future research should focus on: (1) Long-term field performance monitoring of basalt fiber-reinforced recycled mixtures under real traffic and environmental conditions. (2) A comprehensive life-cycle cost–benefit analysis to quantify the economic viability of using basalt fibers in recycled asphalt mixtures. (3) The synergistic effects investigation of basalt fibers with other modifiers (e.g., SBS polymer) to further optimize performance. (4) Micro-scale investigation using techniques such as Scanning Electron Microscopy (SEM) to directly reveal the fiber–asphalt mastic interfacial bonding behavior and reinforcement mechanisms.

## Figures and Tables

**Table 1:** Test results of performance indicators of the extracted aged asphalt.

Technical Indicators	Results	Requirements in [19]
Penetration (25 °C)/0.1 mm	39	50–80
Softening point/°C	69	≥60
Ductility (5 °C)/cm	7.8	≥30
Rotary Viscosity (135 °C)/Pa·s	2.33	≤3

**Table 2:** The gradation of the aggregates in the RAP.

Passing Ratio/%		Sieve Size/mm									
		16	13.2	9.5	4.75	2.36	1.18	0.6	0.3	0.15	0.075
SMA-13	Upper limit	100	100	75	34	26	24	20	16	15	12
	Lower limit	100	90	50	20	15	14	12	10	9	8
RAP aggregates		100	92.1	61.2	31.0	25.4	21.3	16.9	13.3	11.0	8.1

**Table 3:** Performance results of chopped Basalt fibers.

Test Items	Test Results	Requirements in [22]
Density/g-cm-3	2.72	2.60–2.80
Tensile strength/MPa	3800	≥2000
Elongation at break/%	2.69	≥2.1
Elasticity Modulus/GPa	102	≥80
Heat resistance, retention of breaking strength/%	92	≥85
Alkali resistance, retention of breaking strength/%	87	≥75

**Table 4:** Performance Results of lignin fibers.

Test Items	Test Results	Requirements in [19]
Ash content/%	19.3	18 ± 5
pH value	7.7	7.5 ± 1.0
oil absorption	6.2	5 times by the fiber mass

**Table 5:** Design results of six types of mixture proportions.

No.	Gradation Type	Fiber Type	Fiber Content/%	Optimum Oil/Stone Ratio/%
1	Hot mix Asphalt (HMA)	lignin fiber	0.3	6.0
2		basalt fiber	0.3	5.8
3	Hot central plant recycling mixture (HCPR)	lignin fiber	0.3	6.1
4		basalt fiber	0.3	5.9
5	Hot in-place recycling mixture (HIPR)	lignin fiber	0.1	6.0
6		basalt fiber	0.3	6.0

**Table 6:** Test results of three major indicators of recycled asphalt.

Pilot Project	Rejuvenator Content/%					SBS Asphalt
	0	4	6	8	10	
penetration (25 °C)/0.1 mm	39	60	68	74	78	71
Softening point/°C	69	65	63	61	56	64
Ductility (5 °C)/cm	7.8	22.4	28.6	31.4	34.6	48

**Table 7:** Results of Dynamic creep test.

Type of Mix	Stage II Model	R^2^	Creep Rate	Rheological Number Fn
HMA + lignin	y = 46x + 17,778	0.993	46	552
MHA + BF	y = 32x + 16,984	0.992	32	748
HCPR + lignin	y = 28x + 20,166	0.983	28	782
HCPR + BF	y = 25x + 27,159.5	0.997	25	963
HIPR + lignin	y = 22x + 21,747	0.983	22	980
HIPR + BF	y = 17x + 28,341	0.992	17	1289

**Table 8:** Results of Hamburg rutting test.

Mix Type	Maximum Rutting Depth/mm	Creep Slope	Spalling Slope	Spalling Point/Time
HMA + lignin	6.91	1.3 × 10^−4^	/	/
MHA + BF	5.71	8.7 × 10^−5^	/	/
HCPR + lignin	8.15	1.8 × 10^−4^	4.0 × 10^−4^	12,483
HCPR + BF	6.94	1.5 × 10^−4^	3.1 × 10^−4^	15,630
HIPR + lignin	10.03	2.6 × 10^−4^	5.8 × 10^−4^	8637
HIPR + BF	9.07	2.2 × 10^−4^	5.2 × 10^−4^	10,536

## Data Availability

The original contributions presented in this study are included in the article. Further inquiries can be directed to the corresponding author.
